# Recent Progress in Adsorption Removal of Heavy Metal Ions from Wastewater Using Biomass-Based Materials

**DOI:** 10.3390/gels12040311

**Published:** 2026-04-05

**Authors:** Chenxi Sui, Wantong Xie, Yujing Bian, Xiang Li

**Affiliations:** 1 College of Textiles and Apparel, Keyi College of Zhejiang Sci-Tech University, Shaoxing 312000, China; 19588233137@163.com (C.S.); 18006596386@163.com (W.X.); 2 College of Materials and Environmental Engineering, Hangzhou Dianzi University, Hangzhou 310012, China

**Keywords:** biomass-based materials, adsorption properties, adsorption mechanism, heavy metal ions

## Abstract

Heavy metal pollution poses a serious threat to water resource security and ecological health, due to its high toxicity, persistence, and bioaccumulation. Accordingly, it is crucial to develop efficient, low-cost, and environmentally friendly adsorption materials. Biomass-based materials, as a widely available, renewable, and low-cost natural organic resource, exhibit significant advantages for water pollutant adsorption and removal due to their unique porous structures and abundant active functional groups. This review systematically summarizes the classification strategies, fabrication methodologies, and adsorption performances of biomass-based materials for aqueous heavy metal ion removal. Key factors governing adsorption behavior, including solution pH, temperature, initial ion concentration, and adsorbent dosage, are critically analyzed to elucidate structure–property–performance correlations. Particular emphasis is placed on the underlying adsorption mechanisms, encompassing physical adsorption, surface complexation, ion exchange, electrostatic interactions, and synergistic interfacial effects. By integrating recent advances in material design and mechanistic understanding, this review provides a comprehensive framework bridging fundamental research and practical implementation, and highlights future opportunities for engineering next-generation sustainable biomass adsorbents toward efficient heavy metal ion decontamination.

## 1. Introduction

With the accelerated advancement of global urbanization and industrialization, the multiple pollution sources from industrial production emissions, agricultural non-point source diffusion, and urban domestic sewage leakage have increasingly highlighted water resource pollution. Water pollution has become a global ecological and environmental challenge, threatening ecosystem balance and human survival and health. Among them, heavy metal ions, as a typical category of persistent pollutants, exhibit characteristics such as strong toxicity, difficult degradation, bioaccumulation, and mobility [1,2]. Once released into water bodies, heavy metal ions are persistent and difficult to be degraded by natural ecological processes [3]. Thus, heavy metal ions are ingested and accumulated by aquatic organisms, ultimately entering the human body via the food chain. This leads to a series of health risks to human beings, such as bone damage, organ pathology, nervous system disorders, and genotoxicity [4,5,6]. The World Health Organization (WHO) explicitly stipulates in drinking water quality standards that the limits for Pb^2+^, Cd^2+^, and Hg^2+^ in drinking water are 0.01 mg L^−1^, 0.001 mg L^−1^, and 0.001 mg L^−1^, respectively, underscoring the urgency and stringent requirements for controlling heavy metal ion pollution in water bodies. Therefore, effectively removing heavy metal ions from wastewater is vital for protecting human health and improving environmental quality.

Currently, various technical approaches have been developed to address heavy metal ion pollution in wastewater, including chemical precipitation [7,8], precipitation [9], ion exchange [10,11], membrane separation [12,13,14], and electrolytic reduction [15]. These conventional methods are hindered by the balance of treatment efficiency, cost, and environmental sustainability. In contrast, adsorption technology has emerged as one of the most promising solutions due to its high removal efficiency, broad applicability, and simple operation. However, adsorption technology still remains a challenge in fabricating adsorbent materials and generating secondary pollution. Therefore, exploring efficient, cost-effective, and sustainable adsorbent materials has become a hot topic in addressing heavy metal pollution.

With the awareness of environmental protection and the promotion of sustainable development, heavy metal ion adsorption materials have shifted from traditional inorganic, organic, and composite materials to biomass materials. As an abundant, renewable, and cost-effective natural resource, biomass materials demonstrate potential application in the adsorption field. Biomass-based materials primarily originate from plants (especially crop residues), animals, and microorganisms. Biomass-based materials contain sufficient active functional groups, such as hydroxyl, carboxyl, amino, or thiol groups, in their molecular structures, as well as having a hierarchical porous structure. These features enable biomass-based materials to effectively capture heavy metal ions in water through complexation, ion exchange, and physical adsorption. Meanwhile, biomass-based materials are biodegradable, resulting in a low environmental impact, aligning with sustainable development [16,17].

Herein, this review offers a systematic overview on the classification, structural characteristics, preparation methods, adsorption mechanisms, and current applications of biomass-based materials, thereby paving a theoretical foundation for the design of sustainable biomass-derived adsorbents in heavy metal ion removal. Compared with existing studies, particular attention is given to the correlation between material structure and adsorption performance, as well as the interplay of multiple adsorption mechanisms under different conditions. By integrating recent advances in material design and mechanistic insights, this review provides a more coherent framework between fundamental understanding and practical application. The reviewed technological advancement and engineering development of biomass-based materials not only overcome the limitations of conventional water treatment technologies but also enable the high-value utilization of agricultural and forestry waste. Therefore, this review contributes to resource recycling and advances the realization of the sustainable development goals.

## 2. Biomass-Based Heavy Metal Ion Adsorption Materials

### 2.1. Plant-Based Biomass Heavy Metal Ion Adsorbent

Plant-based biomass materials are one of the most abundant resources in nature, including straw, sawdust, rice husks, and corn cobs, which consist of cellulose, hemicellulose, and lignin. They contains hydroxyl groups and related functional groups in their molecular chains. These groups can adsorb heavy metal ions in water through hydrogen bonding, complexation, and other interactions.

#### 2.1.1. Straw-Derived Heavy Metal Ion Adsorbent

**Figure 1:**
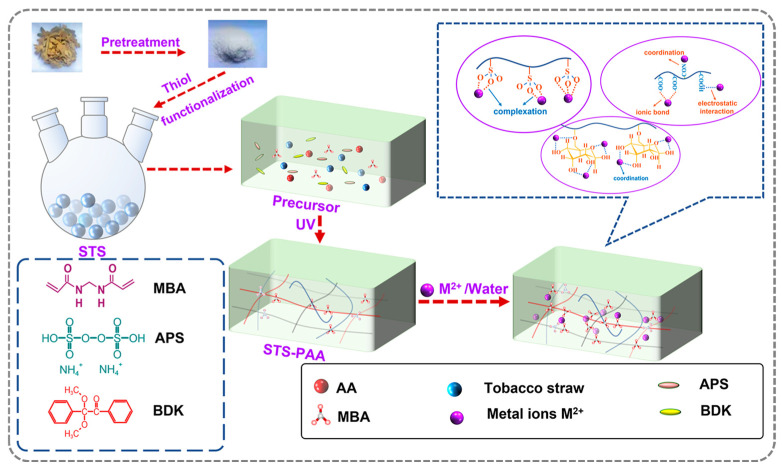
Synthesis and adsorption mechanism for STS-PAA (the lines with different colors in the STS-PPA hydrogel are represented by sulfhydryl modified tobacco straw, PAA, and STS-PPA molecular chains) [18]. Adapted from Ref. [18] (an open-access article distributed under the terms of the CC BY).

Straw is the stalk part left behind after grain harvest. The main components of straw are cellulose, hemicellulose, and lignin. The majority of the hydroxyl groups in the cellulose molecular chain are the active sites for heavy metal ion adsorption, which capture heavy metal ions through the formation of coordination or hydrogen bonding. For instance, Zhang et al. [18] prepared a porous tobacco straw-based polyacrylic acid (STS-PAA) hydrogel by UV radiation-induced polymerization of acrylic acid/potassium acrylate with pretreated waste tobacco straw (Figure 1). The resulting STS-PAA hydrogel showed high adsorption efficiency, with equilibrium adsorption capacities for Pb^2+^, Cd^2+^, and Hg^2+^ of 1.49 mmol g^−1^, 1.02 mmol g^−1^, and 0.94 mmol g^−1^, respectively, at pH = 6 and initial concentration of 4.0 mmol L^−1^. Zhong et al. [19] prepared humic acid (HS) and aerobic humic substances (AE-HSs) extracted from wheat straw and investigated their adsorption performance for heavy metals in contaminated soil, including cadmium (Cd), copper (Cu), zinc (Zn), and lead (Pb). Notably, the optimized AE-HS achieved the highest removal efficiencies of 96.18%, 82.75%, 60.43%, and 41.66% for Cd, Cu, Zn, and Pb, respectively. The AE-HS was obtained by effective aerobic treatment, which promotes the degradation of straw to form high-concentration, low-molecular-weight, and highly humified AE-HSs. Therefore, the AE-HS is rich in phenolic compounds, carboxylic acids, and active groups such as hydroxyl, carboxyl, and carbonyl, which significantly enhance the adsorption capacity for heavy metals. These studies demonstrate that straw-based biomass can effectively adsorb heavy metal ions through porous structure design or molecular functionalization. Straw-derived biomass materials serve as efficient and sustainable adsorbents for heavy metal removal from water.

#### 2.1.2. Rice Husk-Derived Heavy Metal Ion Adsorbent

Rice husks are the outer shells of rice seeds, and are composed of unique silica, cellulose, and lignin. The existence of amorphous silica endows the rice husk with a rigid framework and excellent structural stability. Simultaneously, the silanol groups (-Si-OH) in their molecular structures can produce stable Si-O-M bonds with heavy metal ions (where M represents heavy metal ions), resulting in the enhancement in adsorption stability. Hinsene et al. [20] fabricated structurally stable, recyclable ternary composites (Fe_3_O_4_-ZnO/RBC-DES) by combining rice husk-derived biochar doped with deep eutectic solvent and Fe_3_O_4_/ZnO nanoparticles. The as-prepared material exhibited outstanding adsorption performance for Cr(VI) and Pb(II), with maximum adsorption capacities of 66.23 mg g^−1^ and 384.6 mg g^−1^, respectively, confirming its potential for heavy metal removal and resource utilization. Liu et al. [21] developed an eco-friendly straw-derived adsorbent (WS-CA-AM) by grafting with acrylamide (AM) and citric acid (CA) groups to remove heavy metals from an aqueous solution. The adsorption capacities of WS-CA-AM for Cr_2_O_7_^2−^ and Cu^2+^ reached 196% and 151%, respectively, which are attributed to the synergistic mechanism involving electrostatic attraction and surface complexation with the functional groups. This result indicates that WS-CA-AM is a potential applicant for the removal of heavy metal ions from mixed aqueous solutions. Sharma et al. [22] developed a chitosan–dicalcium–hemicellulose aerogel with a three-dimensional porous structure by oxidizing rice husk-derived hemicellulose into dicalcium–hemicellulose. The obtained aerogel showed excellent adsorption performance for trivalent arsenic ions (As(III)), with a maximum adsorption capacity of 185 μg g^−1^. The adsorption removal rate of As(III) reached over 98% under optimized conditions. Rice husk-derived adsorbents play a significant role in heavy metal remediation. Therefore, rice husk-derived materials have inherent structural stability determined by the amorphous silica framework, as well as abundant active sites for heavy metal complexation. This synergy between physical robustness and chemical functionality makes rice husk-derived materials an ideal candidate for designing advanced adsorbents for water remediation.

#### 2.1.3. Corn Cob-Derived Heavy Metal Ion Adsorbent

Corn cob is obtained from the residual core of corn after dehulling, which is mainly composed of hemicellulose and cellulose. Corn cobs have inherent loose and porous structures, endowing them with natural adsorption sites. The hemicellulose in corn cob can be hydrolyzed into xylose under acidic conditions, facilitating the formation of hydroxyl and aldehyde groups. Among these functional groups, the aldehyde groups are capable of forming chelates with heavy metal ions, further enhancing the adsorption capacity.

To further improve the adsorption performance, various physicochemical modification strategies have been employed to modify the corn cob-derived adsorbent. For instance, Feng et al. [23] prepared a dual-network hydrogel using sulfonated corn stalks (SCSs) as a starting material and acrylic acid as a crosslinker. The fabricated SCS gel achieved higher mechanical strength than that of the SCS without crosslinking. Outstanding adsorption performance was maintained over wide pH range (2.0–6.0); the maximum adsorption capacities for Pb^2+^ and Cu^2+^ reached 111.6 mg g^−1^ and 370.2 mg g^−1^, respectively, with an efficiency approaching 100% after 10 adsorption–desorption cycles. These results indicate the excellent reusability of the SCS gel. It was revealed that multiple functional groups (–OH, C-O, C=O, COOH, and −SO^3−^) participated in the adsorption process through coordination and electrostatic interactions with the heavy metal ions. In practical mine wastewater purification, removal rates of 95.7–99.8% for various heavy metal ions were achieved by the SCS gel at a dosage of 5 g L^−1^, demonstrating its potential application.

In addition to the corn cob-derived hydrogel, the aerogel also showed high performance in heavy metal ion removal. For example, Luo et al. [24] synthesized a friendly and inexpensive biosorbent through the incorporation of corn cob-derived biocarbon aerogel powders with nanoscale TiO_2_ (TD) via ultrasonic treatment. The resulting composites showed simultaneous removal of Cd(II) and As(V) from non-ferrous metal tailings with maximum adsorption capacities of 72.62 mg g^−1^ and 118.06 mg/g, respectively. Furthermore, functionalization of corn cob-derived materials has been explored to expand their application scope. For example, Wang et al. [25] successfully prepared a dimercapto-modified cellulose hydrogel (SCDs-KTOCS gels) using corn cob as the raw material, enabling simultaneous detection and adsorption of Hg(II). The results showed that the adsorption capacity of SCDs-KTOCS gels for Hg(II) reached 193 mg g^−1^. Meanwhile, a good linear relationship between adsorption intensity and Hg(II) concentration was observed within the range of 150–500 mg L^−1^. Overall, it is demonstrated that corn cob-derived materials can be effectively engineered into high-performance adsorbents through rational structural design and functional modification. Enhanced adsorption capacity, structural stability, and recyclability can thus be achieved.

#### 2.1.4. Sawdust-Derived Heavy Metal Ion Adsorbent

Sawdust is one of the byproducts generated during wood processing and has an intrinsic porous structure. Cellulose and lignin are the main components in sawdust. In particular, lignin contains aromatic ring structures that enable π–π interactions with organic pollutants, while surface functional groups such as hydroxyl and methoxy groups provide active sites for binding with heavy metal ions, thereby contributing to its adsorption capability.

**Figure 2:**
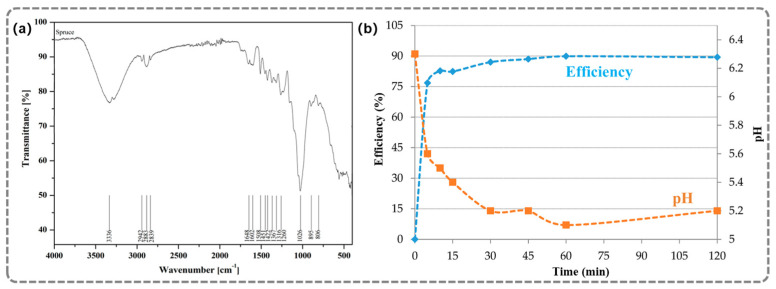
(**a**) Infrared spectrum of spruce sawdust. (**b**) Dependence of sorption efficiency η and changes in pH on time during experiment [26]. Adapted from Ref. [26] (an open-access article distributed under the terms of the CC BY).

The adsorption performance of sawdust-derived materials has been systematically investigated. As shown in Figure 2, Kovacova et al. [26] used spruce wood chips with particle sizes of approximately 2 mm as adsorbents and achieved a removal rate for Cu^2+^ exceeding 75%. Rapid adsorption kinetics were observed, with most copper ions being removed within 5 min. During a 24 h adsorption process, the solution’s pH decreased from 6.3 to 5.3, indicating that adsorption and ion exchange processes were involved. These results demonstrate that wood-derived materials can serve as low-cost and efficient adsorbents for Cu^2+^ removal from wastewater. To further enhance functionality, lignin-based composites have been developed. For example, Liu et al. [27] proposed a lignin-based metal composite (MLC) with excellent light absorption and photothermal conversion properties. The composite showed a daily water purification capacity of 17.5 L m^−2^ and a water evaporation rate of 2.88 kg m^−2^ h^−1^ under 1-sun irradiation, demonstrating outstanding performance in both seawater and heavy metal-containing wastewater systems. In addition, sawdust-derived aerogel adsorbents have also been explored. He et al. [28] prepared an aerogel membrane from natural lightwood as the raw material through delignification, TEMPO oxidation and mechanical compression. The adsorption capacity of the aerogel reached 115 mg g^−1^ for Cu(II). The mechanism was possibly attributed to the designed carboxyl groups of the aerogel’s membranes, which enabled strong chelation with Cu(II) ions. Overall, it is demonstrated that sawdust-derived materials can be effectively engineered into high-performance adsorbents through structural modulation and functionalization. The synergistic effects of porous architecture and surface chemistry enable efficient heavy metal removal, while the integration of additional functionalities, such as photothermal conversion, further broadens their application potential in sustainable water treatment.

#### 2.1.5. Bark-Derived Heavy Metal Ion Adsorbent

**Figure 3:**
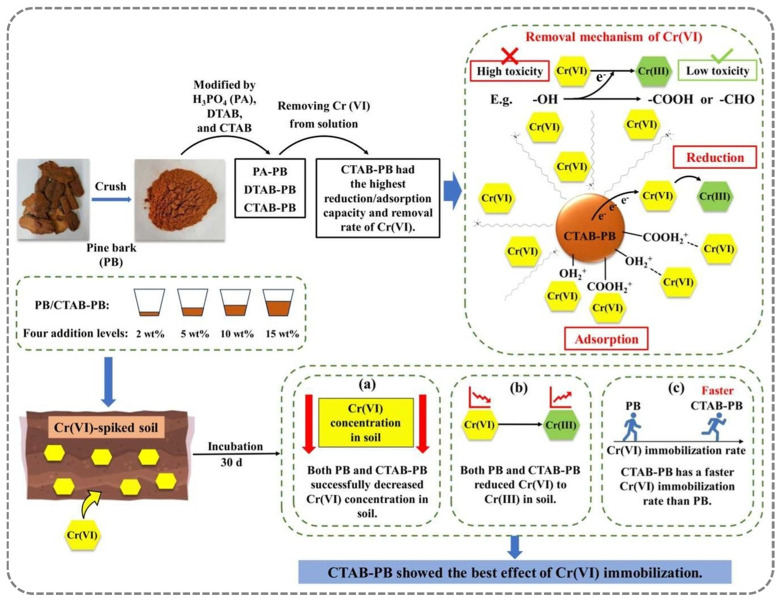
Preparation of the modified pristine pine bark (PB) materials and their absorption mechanisms [31]. Adapted from Ref. [31] (an open-access article distributed under the terms of the CC BY).

Bark, serving as the outer protective layer of trees, is enriched with tannins, lignin, and cellulose, which collectively endow it with abundant functional groups and inherent chemical reactivity [29,30]. For instance, pristine pine bark (PB) was modified using phosphoric acid (PA-PB), dodecyl trimethylammonium bromide (DTAB-PB), and acetyl trimethylammonium bromide (CTAB-PB) to regulate surface charge characteristics and adsorption affinity [31]. Among them, CTAB-modified pine bark (CTAB-PB) exhibited the highest adsorption capacity toward Cr(VI), reaching 88.1 mg g^−1^ under optimal conditions in aqueous systems (Figure 3). This enhancement can be attributed to the introduction of cationic surfactant groups, which facilitate electrostatic attraction toward anionic Cr(VI) species, thereby improving adsorption performance.

Furthermore, thermochemical conversion has been employed to further optimize the structural and chemical properties of bark-derived materials. For example, Cheng et al. [32] used poplar sawdust as the raw material to produce a biochar aerogel through pyrolysis at 400–800 °C, which is used as the sustainable adsorbent to investigate the adsorption performance of Pb^2+^/Cd^2+^ from wastewater. The maximum adsorption capacities of the biochar aerogel reached 62.68 mg/g for Pb^2+^ and 49.32 mg g^−1^ for Cd^2+^ at pH = 5. Mechanism analysis revealed that Pb^2+^ removal was predominantly governed by mineral precipitation, whereas Cd^2+^ adsorption was primarily associated with coordination interactions involving π-electron systems. These studies demonstrate that bark-derived biomass can be rationally engineered through surface modification and thermochemical processing to achieve tunable adsorption behavior. The surface functionality, charge characteristics, and microstructure are identified as the key determinants for achieving high efficiency and selectivity in heavy metal ion removal, offering valuable insights for the design of next-generation biomass-based adsorbents.

### 2.2. Animal-Based Biomass Heavy Metal Ion Adsorbents

Animal-derived biomaterials include wool, silk, eggshell, fish scale, shrimp, and crab shells, etc. The main components are protein, calcium carbonate, and chitin. The existing functional groups, such as amino and carboxyl, are capable of interacting with heavy metal ions through coordination, electrostatic attraction, and ion-exchange mechanisms.

#### 2.2.1. Protein-Derived Heavy Metal Ion Adsorbent

Wool and silk are representative protein-based natural fibers. Among them, wool is rich in keratin, which contains abundant functional groups, including amino (-NH_2_), carboxyl (-COOH), hydroxyl (-OH), and thiol (-SH) [33]. These groups act as active binding sites for heavy metal ions via coordination and chelation. Notably, thiol groups demonstrate outstanding binding capacity towards heavy metals such as As, Pb, and Ca [34]. To exploit these molecular-level interactions, keratin-based materials have been further engineered through hybridization and crosslinking strategies. For example, Zubair et al. [35] prepared biosorbents for water remediation using a keratin biopolymer crosslinked with nanochitosan (NC). The biosorbents showed a high adsorption capacity of up to 98% for multiple metal ions, including arsenic, selenium, chromium, nickel, cobalt, lead, cadmium, and zinc, at pH 7.5 and a contact time of 24 h. This enhanced performance can be attributed to the synergistic effect between keratin functional groups and the additional binding sites provided by chitosan.

**Figure 4:**
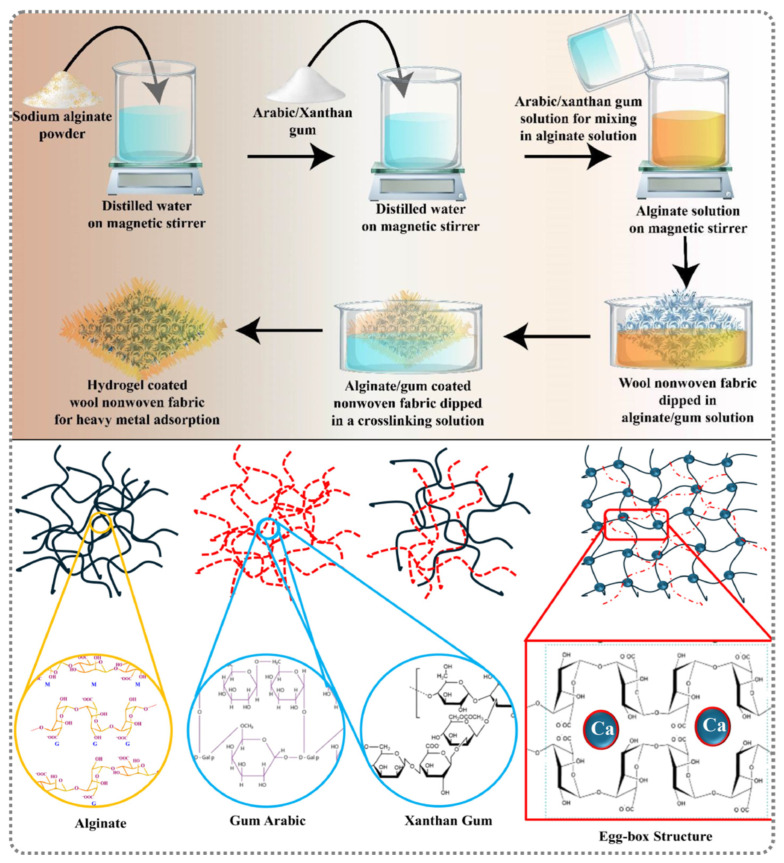
Schematic diagram of the development the alg/GA and alg/XG hydrogel composite reinforced by wool fabric [36]. Adapted from Ref. [36] (an open-access article distributed under the terms of the CC BY).

In addition, composite hydrogel systems have been developed to further regulate adsorption behavior. Azam et al. [36] synthesized a hydrogel adsorbent using wool nonwoven fabric combined with alginate (alg), gum arabic (GA), and xanthan gum (XG) to evaluate the effect of the adsorption of Pb from aqueous solutions (Figure 4). The hydrogel demonstrated the maximum adsorption of 85.2 mg g^−1^ under alkaline pH conditions. Kinetic studies indicated that the adsorption process followed a pseudo-second-order model. Isotherm fitting using the Freundlich model (R^2^ = 0.95) suggested that chemisorption dominated the adsorption mechanism. Beyond conventional heavy metal removal, keratin-based aerogel materials have also been explored for high-value metal recovery. For instance, Chen et al. [37] successfully fabricated wool keratin/cellulose composite aerogels through ether-functionalized proton ion liquid co-dissolution, sol–gel transition, and freeze-drying technologies. The optimized aerogel exhibited high adsorption capacities of 330.8 mg g^−1^ for Au(III) in an aqueous solution and 322.4 mg g^−1^ in simulated electronic wastewater. Moreover, the recovery efficiency of up to 94% was retained after five cycles, indicating excellent reusability and stability. These advancements demonstrate that wool-derived keratin materials can be rationally engineered into versatile adsorption platforms through molecular design and structural integration. The combination of abundant functional groups, tunable network structures, and synergistic interactions enables not only efficient heavy metal ion removal but also selective recovery of high-value metal ions, highlighting their broad application potential in sustainable water treatment and resource recovery.

Silk fibroin is degummed from silk and has been widely employed as a protein-based biosorbent due to its well-defined molecular structure and abundant functional groups. The presence of amino, carboxyl, and hydroxyl groups enables effective interactions with heavy metal ions through coordination and electrostatic interactions, thereby facilitating adsorption performance. For instance, Pilley et al. [38] prepared a powdered silk fibroin (SF) adsorbent using silk as the starting material. The maximum adsorption capacity of the SF for Fe^3+^ reached 12.82 mg g^−1^, and it had a removal efficiency of up to 98% under conditions of 0.25 g adsorbent dosage, 60 min, and pH 6–10. The adsorption kinetics followed a pseudo-second-order model, and the isotherm fitted a Langmuir model, indicating single-layer chemical adsorption. The SF adsorbent was reusable up to five times and demonstrated stable environmental compatibility.

To further enhance adsorption performance, functionalization and hybridization strategies have been introduced. For example, Shilpa et al. [39] produced silk-magnetic nanocomposite films (SMNCs) by casting silk fibroin (SF) with iron oxide (Fe_3_O_4_) nanoparticles (NPs). The nanocomposites showed a high adsorption capacity of 130 mg/g for Ni^2+^ ions in an aqueous solution. The adsorption followed pseudo-second-order kinetics (R^2^ = 0.998). Similarly, Han et al. [40] developed a dopamine-modified silk fibroin aerogel powder through dopamine in situ polymerization modification. The sample showed maximum adsorption capacities of 115.8, 60 and 60 mg g^−1^ for Cu^2+^, Cd^2+^, and Ni^2+^ heavy metal ions, respectively. The adsorption mechanism of Cu^2+^ originated from the Langmuir-type adsorption isotherm model and the pseudo-second-order kinetic model, due to the additional catechol functional groups introduced during dopamine polymerization.

**Figure 5:**
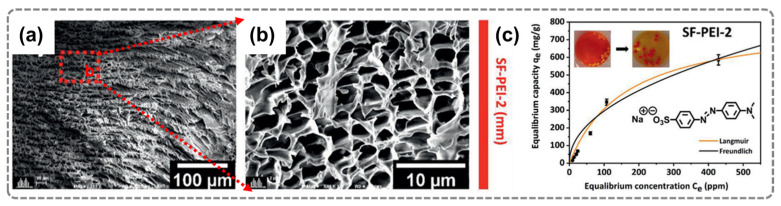
(**a**,**b**) SEM micrographs and (**c**) organic solvent adsorption performance of SF-PEI-2 spheres [41]. Adapted from Ref. [41] (open-access articles distributed under the terms of the CC BY).

Structure design is another strategy to enhance the adsorption performance of silk-based adsorbents. Silk aerogels with hierarchical porous structures have been developed to maximize the surface area and accessibility of active sites. For example, Brud et al. [41] modified the surface of silk fibroin (SF) with polyethylene imine (PEI) and hybridized it with graphene oxide through ice-templating and freeze-drying. The resulting aerogel had millimeter-scale honeycomb-like microporous structures (Figure 5a,b). Benefiting from these features, the aerogel demonstrated excellent adsorption capacity for Cu^2+^ (Figure 5c). In addition, Li et al. [42] prepared silk fibroin/chitosan (SNF/CS) aerogels through degumming, calcium salt soaking, and freeze-drying. These aerogels showed excellent adsorption capacities for Cu^2+^, Ni^2+^, and Cd^2+^, with values of 10.2, 21.3, and 19.2 mg g^−1^, respectively. The corresponding removal rates for Cu^2+^, Ni^2+^, and Cd^2+^ were maintained at 97.5%, 95.5%, and 96.5%, respectively, after three filtrations. Therefore, silk fibroin-based materials can be effectively engineered through structural design and surface functionalization to achieve enhanced adsorption performance. The combination of tunable architectures and diverse functional groups enables efficient removal of heavy metal ions and provides a versatile platform for advanced water treatment applications.

#### 2.2.2. Chitin-Derived Heavy Metal Ion Adsorbent

**Figure 6:**
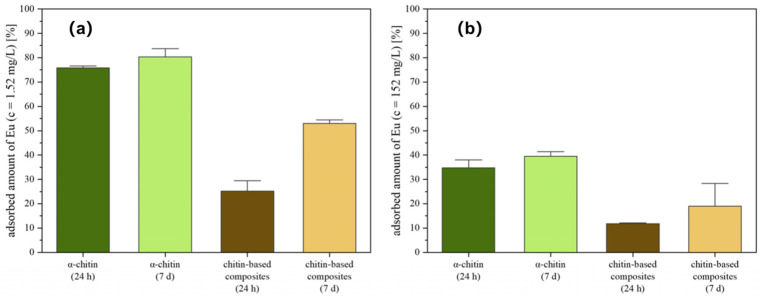
Characterization of the europium adsorption on chitin and chitin-based composites by ICP-OES: (**a**) adsorbed europium amount after 24 h and 7 d [c(EuCl_3_∙6H_2_O) = 10^−5^ M] and (**b**) adsorbed europium amount after 24 h and 7 d [c(EuCl_3_∙6H_2_O) = 10^−3^ M] [45]. Adapted from Ref. [45] (an open-access article distributed under the terms of the CC BY).

Chitin has attracted increasing attention due to its abundance in nature, and is derived from crustacean shells. Chitin (1→4)-2-acetamido-2-deoxy-β-d-glucan intrinsically contains abundant acetyl groups and a nanofibrous architecture with a high specific surface area. Although the degree of deacetylation is relatively low, functional groups such as amino and acetyl groups (-NHCOCH_3_) can bind with heavy metal ions through hydrogen bonds and coordination [43]. Meanwhile, the interconnected chitin nanofiber network structures have a high specific surface area, which can increase the contact sites with heavy metal ions and improve the adsorption efficiency. For example, Shi et al. [44] investigated the preparation of spherical-like chitin-derived carbon adsorbents (SA@DE/CBC) using natural diatomite (DE), biochar (CBC), and sodium alginate (SA) as raw materials for Pb(II) removal (Figure 6). The results showed that SA@DE/CBC conforms to the Langmuir model, with maximum single-layer adsorption capacities reaching 1155 mg g^−1^. The high capacity is attributed to the synergistic effect of the porous structure and functional group interactions. In addition, chitin-based composite gels were prepared for Eu^3+^ removal, achieving an adsorption capacity of 38 mg g^−1^ at a high concentration [45]. The adsorption process was governed by the coordination of carbonyl groups and hydrogen bonding, while the structure of the hydrogel was well preserved after 7 days of adsorption treatment. Overall, chitin-derived biomaterials exhibit distinct adsorption mechanisms depending on their composition. Chitin-based materials benefit from functional group interactions and hierarchical structures. Such complementary characteristics provide a versatile platform for designing efficient and sustainable adsorbents for heavy metal remediation.

### 2.3. Microorganism-Based Biomass Heavy Metal Ion Adsorbents

#### 2.3.1. Bacteria- and Fungus-Derived Heavy Metal Ion Adsorbent

Microorganism-derived biomass, particularly those from bacteria and fungus, contains intrinsic bioactivity and diverse surface chemistry. The cell walls of these organisms are rich in polysaccharides and proteins, providing abundant functional groups such as phosphate, carboxyl, amino, and carbonyl moieties, which can serve as active binding sites for heavy metal ions. The adsorption of heavy metal ions by microorganisms is governed by multi-pathway synergistic mechanisms. Notably, compared with plant- and animal-derived biomass, microorganism-based materials exhibit higher functional diversity and can additionally enable redox transformation of heavy metal ion species [46]. Metal ions can be immobilized through complexation and chelation with surface functional groups, while certain microbial species are capable of transforming toxic metal ions into less toxic forms via redox processes [47]. This combination of physicochemical adsorption and biological transformation enables enhanced removal efficiency and reduced environmental risk.

**Figure 7:**
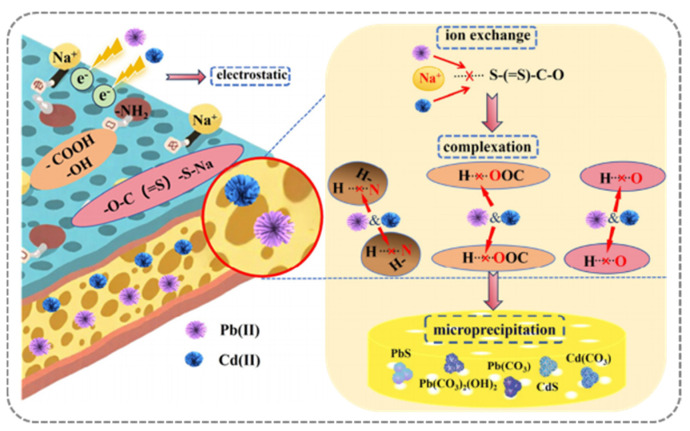
Mechanism diagram of Pb(II) and Cd(II) adsorption on XBS [49]. Adapted from Ref. [49] (an open-access article distributed under the terms of the CC BY).

To leverage these advantages, hybrid systems integrating microbial activity with engineered structures have been developed. For example, cadmium-resistant bacterial strains were combined with sludge-derived biochar to form composite adsorbents, achieving a Cd^2+^ adsorption capacity of 79.97 mg g^−1^ [48]. The enhanced performance is attributed to the synergistic effect between the porous structure of biochar and the metabolic activity of microorganisms. Fungal-derived functional materials have also demonstrated high-efficiency heavy metal ion adsorption. A xanthate-modified adsorbent (XBS) derived from *Phanerochaete sordida* YK-624 was constructed as a three-dimensional porous structure for Pb^2+^ and Cd^2+^ removal [49]. The results showed that XBS achieved 96% Pb(II) and 32% Cd(II) removal within 1 min at a 0.25 g L^−1^ dose, reaching over 95% of the maximum adsorption capacity within 30 min for Pb(II) and 240 min for Cd(II). The maximum capacities were 224.72 mg g^−1^ for Pb(II) and 82.99 mg g^−1^ for Cd(II). The removal mechanism involved ion exchange, complexation, and precipitation, leading to the formation of stable compounds such as PbS/CdS and PbCO_3_/CdCO_3_ (Figure 7).

In addition, bacterial biomass-derived aerogels have been explored to further enhance adsorption performance. A polydopamine-functionalized bacterial cellulose aerogel (PDA-BC) with a three-dimensional porous network was developed for Cr(VI) removal [50]. Benefiting from its interconnected structure and abundant functional groups, a removal efficiency of up to 99% was achieved. Hence, microorganism-based materials exhibit unique advantages by integrating surface functional group interactions with biological transformation pathways. The combination of structural design and bioactivity provides an effective strategy for developing high-performance and sustainable adsorbents for heavy metal ion remediation.

#### 2.3.2. Algae-Derived Heavy Metal Ion Adsorbent

Algae, as eukaryotic photosynthetic organisms, have emerged as promising biosorbents due to their low cost, wide availability, and environmental compatibility. Their cell walls are mainly composed of polysaccharides and proteins, providing abundant functional groups that can interact with heavy metal ions. The removal of heavy metal ions by algae is generally governed by a two-stage process, namely biosorption and bioaccumulation. Biosorption occurs at the cell surface, where metal ions are immobilized via complexation and electrostatic interactions with surface functional groups. In contrast, bioaccumulation involves the active transport of metal ions into the intracellular region, leading to further enrichment. This dual mechanism enables efficient and sustained removal of heavy metals.

**Figure 8:**
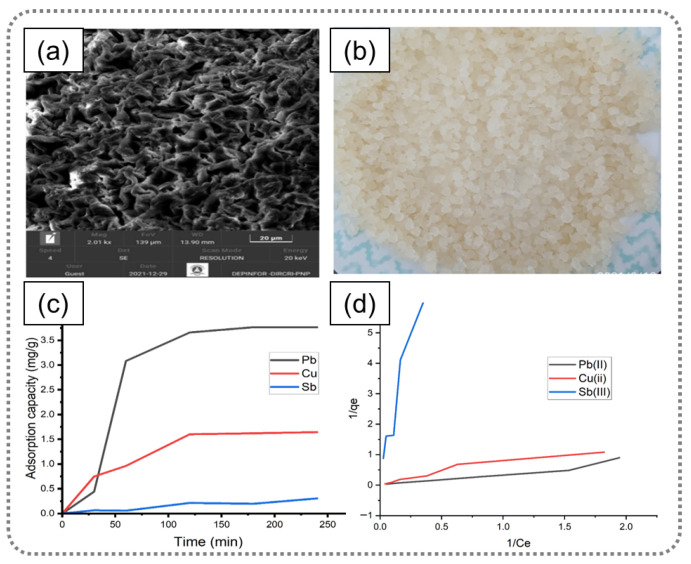
(**a**) SEM microphotography of calcium alginate and (**b**) calcium alginate is 2–3 mm in diameter. (**c**) Removal efficiency of metal ions at the following conditions: recirculation flow of 5 mL/s, adsorbent mass of 10 g, concentration of ions 10 mg/L, and agitation with air. (**d**) Langmuir isotherm applied to the adsorption of Pb(II), Cu(II), and Sb(III) by alginate [51]. Adapted from Ref. [51] (an open-access article distributed under the terms of the CC BY).

Various algae-derived materials and composites have been developed. As shown in Figure 8, calcium alginate beads prepared from brown algae were utilized for Pb^2+^, Cu^2+^, and Sb^3+^ removal, achieving removal efficiencies of 92%, 78%, and 16%, respectively. The maximum adsorption capacities reached 7.60, 2.07 and 0.37 mg g^−1^ under optimized conditions [51]. In addition, green algae-derived biochar was further functionalized with Fe_3_O_4_ and Cu-based metal–organic frameworks to construct a magnetic composite adsorbent [52]. The porous biochar was activated by KOH; thus, a high adsorption capacity of 227.95 mg g^−1^ for Cr(VI) was achieved, with removal governed by ion exchange, electrostatic interaction, redox processes, and lattice capture. Alginate-based composites have also been widely explored for rapid and selective adsorption. For example, Peng et al. [53] obtained a novel crosslinked collagen fibrous hydrogel (OSA@CF/Zr) for ultra-rapid recovery of Sr^2+^. The OSA@CF/Zr showed rapid adsorption of Sr^2+^, with a maximum adsorption capacity of 0.415 mmol g^−1^. Even after 17 cycles of reuse, its adsorption capacity remained approximately 0.26 mmol g^−1^, highlighting its superior reusability. Similarly, a sodium alginate-based composite aerogel demonstrated a high adsorption capacity of 228.24 mg g^−1^ for Cu^2+^, following Langmuir-type isotherm behavior and pseudo-second-order kinetics [54]. The enhanced performance is attributed to the synergistic effects of electrostatic attraction, coordination complexation, and ion exchange. It can be concluded that algae-derived materials combine surface functional group interactions with hierarchical structures and tunable compositions, enabling efficient heavy metal removal. Their versatility in forming beads, biochar, and aerogel systems provides a scalable and sustainable platform for wastewater treatment, particularly in complex multi-ion environments.

## 3. Factors Affecting the Heavy Metal Ion Adsorption Performance

The adsorption efficiency of biomass materials for heavy metal ions is regulated by multiple factors, including solution pH, temperature, and adsorbent dosage. Clarifying the mechanisms of these factors is crucial for optimizing adsorption conditions and enhancing practical application.

### 3.1. Effect of pH

The pH is recognized as a critical factor governing adsorption performance, as it directly affects the surface charge of the adsorbent, the ionization state of adsorbates, and the dissociation of functional groups at active sites. These effects determine the interaction strength between biomass adsorbents and heavy metal ions. In general, improved adsorption of cationic heavy metals is observed under neutral to mildly alkaline conditions. Under acidic environments, excessive H^+^ ions compete with metal ions for active binding sites, leading to suppressed adsorption. As the pH increases, protonation of functional groups is reduced, thereby exposing more binding sites and enhancing electrostatic attraction and coordination interactions. For example, a chitosan-based porous composite (MS/CTS) exhibited progressively enhanced Hg^2+^ adsorption with increasing pH, which was attributed to reduced protonation and diminished electrostatic repulsion [55].

However, the optimal pH is strongly dependent on the speciation of metal ions and the surface chemistry of the adsorbent. In the case of a cellulose-based composite aerogel incorporating metal–organic frameworks, different optimal pH conditions were identified for various metal ions [56]. As a result, pH = 3 is the best condition for adsorbing V(V) and Mo(VI), and pH = 7 is the best condition for adsorbing Ni(II). At pH = 3, vanadium exists mainly as H_3_V_10_O_28_^3−^ and molybdenum as HMoO^4−^, and these complex anions can engage with numerous positively charged functional groups.

It should be noted that excessive pH can induce precipitation effects, which may interfere with the accurate evaluation of adsorption capacity. For instance, the adsorption of Cu(II) by tea waste-derived biosorbents increased with pH up to 5 due to enhanced deprotonation and surface complexation [57]. However, under acidic conditions (pH < 5), the deprotonation of functional groups was intensified, leading to an increase in surface negative charge sites, which significantly enhanced Cu(II) adsorption via the complexation mechanism. A similar trend was observed for Cr^3+^ adsorption using starch-based composite aerogels, where optimal adsorption occurred at pH 5, while higher pH conditions resulted in Cr(OH)_3_ precipitation [58]. pH-dependent adsorption behavior has also been observed in alginate-based adsorbents. Phosphate-functionalized calcium alginate microspheres exhibited distinct optimal pH values for different metal ions, with maximum removal achieved at pH 4.0 for Pb(II) and pH 5.5 for Cd(II) [59]. The enhanced performance was attributed to reduced proton competition at a moderate pH and the formation of stable complexes or precipitates between phosphate groups and metal ions. Therefore, pH influences adsorption through multiple coupled mechanisms, including proton competition, surface charge regulation, metal ion speciation, and precipitation effects. Precise control of solution pH is essential for optimizing adsorption performance and accurately distinguishing between true adsorption and precipitation-driven removal.

### 3.2. Effect of Temperature

**Figure 9:**
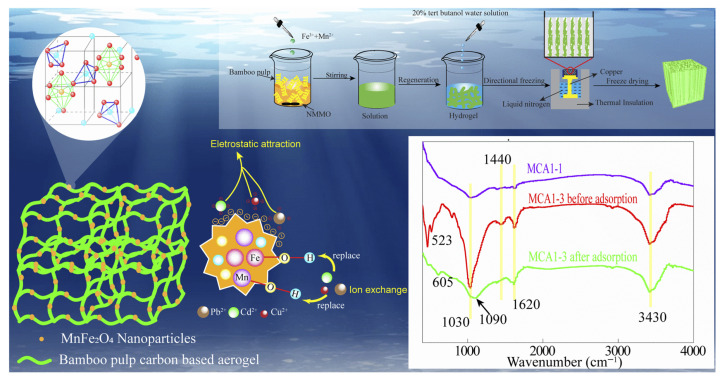
Schematic of the preparation and adsorption mechanism of MnFe_2_O_4_-loaded bamboo pulp carbon-based aerogel (MCA) via directional freeze-drying and carbonization [60]. Adapted from Ref. [60] (an open-access article distributed under the terms of the CC BY).

Temperature is another key factor influencing adsorption behavior, exerting a dual effect on both kinetics and thermodynamics. On the one hand, elevated temperatures enhance molecular motion, thereby accelerating the diffusion of pollutants from the bulk solution to the adsorbent surface and improving mass transfer efficiency. On the other hand, since many adsorption processes are exothermic in nature, increasing temperature may shift the adsorption–desorption equilibrium toward desorption, resulting in reduced adsorption capacity. In practical applications, optimal temperature conditions must be selected based on specific adsorption systems and requirements. For certain biomass-based systems, adsorption capacity has been observed to increase with temperature, indicating an endothermic adsorption process. For example, a sodium alginate/cellulose nanofiber composite hydrogel showed enhanced adsorption capacities for Pb^2+^, Cu^2+^, and Cd^2+^ with increasing temperature [1]. Temperatures not only enhance the diffusion kinetics of heavy metal ions, facilitating their penetration through the liquid film barrier and diffusion to internal adsorption sites, but also activate surface active groups within the hydrogel, thereby improving the reaction rate and extent of complexation with heavy metal ions. Similarly, a bamboo pulp-derived carbon-based composite aerogel loaded with MnFe_2_O_4_ nanoparticles demonstrated temperature-enhanced adsorption performance toward Pb^2+^, Cu^2+^, and Cd^2+^ [60]. The adsorption process is an endothermic spontaneous reaction. Temperature enhances the spontaneity of the reaction (Figure 9). Based on the above analyses, temperature influences adsorption through coupled kinetic and thermodynamic effects, including diffusion enhancement, activation of functional groups, and equilibrium shifts. Therefore, optimization of temperatures is required to balance adsorption efficiency and thermodynamic stability, particularly in complex wastewater systems with multiple heavy metal ions.

### 3.3. Effect of Adsorbent Dosage

Adsorbent dosage is considered an operational parameter influencing adsorption performance. In general, an increase in adsorbent dosage leads to improved removal efficiency, because more active sites become available for pollutant binding. However, beyond a certain threshold, the enhancement in removal efficiency is gradually weakened. This behavior is primarily attributed to two factors. First, excessive adsorbent dosage may induce particle agglomeration, thereby reducing the effective surface area and limiting the accessibility of active sites. Second, at relatively low pollutant concentrations, a portion of adsorption sites remains unoccupied, resulting in decreased utilization efficiency. Consequently, although removal efficiency increases with dosage, the adsorption capacity per unit mass (*qe*) often decreases. In addition, the influence of adsorbent dosage may vary with material size scale, particularly for nanoscale adsorbents, where aggregation and surface energy effects become more pronounced.

This trend has been widely observed in biomass-derived adsorbents. For instance, hemp-derived activated carbon showed a significant increase in the removal efficiency of Ni^2+^ and Cu^2+^ with increasing dosage, followed by a plateau behavior upon the increase in dosage [61]. Despite the adsorption capacity (*qe*) of metal ions decreasing with the increase in the amount of adsorbent, the removal rate of nickel ions can reach 90.89% when the amount of adsorbent is 0.06 g, and the copper ion removal rate can reach 91.21% when the adsorbent amount is 0.05 g. In addition, biomass adsorbents derived from marine brown algae residues showed dosage-dependent adsorption behavior toward Cu(II). As the adsorbent dosage increased, removal efficiency was enhanced due to the increased availability of active sites and reduced competition among metal ions. However, the improvement became less pronounced at higher dosages, indicating the existence of an optimal dosage range [62]. The adsorption process followed a quasi-second-order kinetic model (R^2^ > 0.995), with chemical adsorption as the dominant mechanism.

From an application perspective, the optimization of adsorbent dosage requires a balance between removal efficiency and material cost. Excessive dosage may not lead to proportional performance gains and can reduce economic feasibility. Therefore, identifying an optimal dosage is essential for achieving efficient and cost-effective wastewater treatment.

## 4. Adsorption Mechanism

### 4.1. Physical Adsorption

Physical adsorption is mainly achieved through non-covalent interactions such as van der Waals forces, hydrogen bonds, surface hydrophobic effects, and electrostatic attraction. The porous structure of biomass materials provides a large specific surface area, providing structural support and diffusion channels for the removal of heavy metal ions from water, allowing pollutant molecules to enter the pores via physical diffusion and be adsorbed on the surface, thereby improving overall adsorption efficiency and cyclic stability. Wu et al. [63] successfully synthesized a chitosan (CS)/polyvinylpyrrolidone (PVP)/polyvinyl alcohol (PVA) hollow nanofiber membrane (CS/PVP/PVA-HNM) via coaxial electrospinning with an interwoven nanofiber structure and high porosity for heavy metal (Cu^2+^, Ni^2+^, Cd^2+^, and Pb^2+^) adsorption. Metal ions were efficiently retained on the fiber surfaces and within the pores through intermolecular forces, achieving physical adsorption with retention rates exceeding 85%. Additionally, Zeng et al. [64] prepared magnetic chitosan composites with hydrophobic microdomains and mesoporous structures that can physically retain Cr(VI) and Cu(II) through pore filling and surface hydrophobic interactions. The optimal pHs for Cr(VI) and Cu(II) were 3 and 6, with saturated adsorption capacities of 87.53 and 351.03 mg g^−1^, respectively. According to the findings from Hoang et al. [65], the physical adsorption mechanism of biomass-based activated carbon for heavy metal ions is that they are dispersed and deposited in the pores of the adsorbent through physical action, without the involvement of chemical bonding.

These findings highlight the fundamental role of the pore structure and surface characteristics of the biomass absorbent in governing physical adsorption behavior. Hence, physical adsorption is characterized by its rapid kinetics, structural dependence, and good regeneration potential. However, due to the relatively weak interaction forces, its adsorption capacity and selectivity are generally lower than those of chemisorption, indicating that structural optimization is essential for enhancing its performance.

### 4.2. Chemical Adsorption

Chemical adsorption refers to the formation of chemical bonds or complexes between functional groups on the surface of biomass materials and pollutants (heavy metals) through chemical reactions. For example, the surface of nano-biochar with functional groups such as hydroxyl, carboxyl, and amine groups can form stable complexes with heavy metal ions, including Cd^2+^ and Hg^2+^ [66]. Additionally, electron transfer can reduce Cr^6+^ to Cr^3+^, which is then complexed and immobilized. Chen et al. successfully prepared a novel material (SH@HB/MgFe-LDH) composed of sulfhydryl (SH)-modified hydrothermal biochar (HB) and magnesium–iron layered double hydroxide (MgFe-LDH) [67]. Kinetic and isothermal fitting analyses demonstrated that the arsenic (As) adsorption behavior followed a second-order kinetic model (R^2^ = 0.988) and a Langmuir adsorption isotherm model (R^2^ = 0.976), indicating that the adsorption process was predominantly driven by monolayer chemical adsorption. The efficient removal of arsenic in this composite was primarily achieved through chemical complexation.

### 4.3. Ion Exchange Adsorption

Ion exchange adsorption is achieved through the substitution of ionizable protons or surface-bound cations on biomass materials with heavy metal ions in a solution. In this process, exchangeable ions such as H^+^, Na^+^, K^+^, Ca^2+^, and Mg^2+^ are released into the solution, while target metal ions are immobilized on the adsorbent surface, resulting in effective pollutant removal. For example, the Mg/Fe bimetallic oxide-modified biochar (Fe/Mg-BC) prepared by Cheng et al. was primarily used to adsorb Pb(II) and Cd(II) through an ion-exchange mechanism [68]. The results show that the concentrations of cations such as H^+^, Na^+^, Ca^2+^, and Mg^2+^ in the solution increase during the adsorption process, confirming that Pb(II) and Cd(II) undergo displacement reactions with these exchangeable cations. In the single-metal system, Fe/Mg-BC adsorbs 252.70 mg g^−1^ of Pb(II) and 156.60 mg g^−1^ of Cd(II). In the bimetallic competitive system, the Cd(II) adsorption significantly decreases to 46.44 mg g^−1^, while Pb(II) remains at 220.4 mg g^−1^, indicating that Pb(II) has a competitive advantage due to its larger ionic radius, higher electronegativity, and lower hydration energy. DFT calculations further revealed that the electron cloud overlaps between Pb(II) and the O-site on the MgO surface are denser and orbital hybridization is stronger, thereby explaining its higher affinity for Pb(II) at the electronic level.

In addition, ion exchange often operates synergistically with electrostatic interactions. Zhao et al. [69] prepared a biomass polyamine-functionalized nanocellulose-loaded covalent organic framework composite aerogel (TDB) and investigated its adsorption performance for Cr(VI) in water. The aerogel showed high adsorption capacity for Cr(VI), where positively charged amino groups facilitated electrostatic attraction, followed by ion exchange with surface functional groups. Such a synergistic mechanism enabled high adsorption capacity and selectivity toward target ions. Hence, ion exchange adsorption offers high efficiency, selectivity, and stability, particularly for ionic pollutants. Its performance is governed by the availability of exchangeable ions, competitive interactions in multi-ion systems, and the intrinsic properties of metal ions, highlighting its importance in biomass-based water treatment applications.

### 4.4. Synergistic Adsorption Mechanisms

The adsorption performance of pristine biomass materials is inherently limited by their structural characteristics, as only a restricted number of functional groups (e.g., hydroxyl and carboxyl groups) are typically available as active sites. Although selective adsorption of certain heavy metal ions can be achieved, the overall adsorption capacity is often constrained. To overcome these limitations, synergistic adsorption strategies have been widely developed by integrating multiple functional components or coupling different adsorption mechanisms. In this way, both the diversity and density of active sites can be significantly enhanced, leading to improved adsorption capacity and broadened applicability [70,71,72].

Representative examples highlight the effectiveness of such synergistic designs. Multifunctional aerogels constructed from cellulose and chitosan exhibited enhanced Cu^2+^ adsorption, particularly in multi-component systems [70]. The results showed that in a single-pollution system, the maximum adsorption capacity for copper ions was 202.43 mg g^−1^. In a binary system, the presence of Congo red (CR) and Cu^2+^ mutually enhanced the adsorption efficiency of copper ions to 260.41 mg g^−1^. This phenomenon underscores the importance of interfacial coupling effects in complex wastewater systems. In addition to multi-component adsorption, synergistic mechanisms involving adsorption and redox processes have also been demonstrated. For instance, Zhou et al. investigated the Cr(VI) removal performance of iron-modified biochar, confirming its core mechanism as adsorption–reduction synergy [73]. Cr(VI) was initially enriched on the adsorbent surface via electrostatic attraction and complexation, followed by reduction to the less toxic Cr(III) species by Fe(II), acting as an electron donor. The dual-function process significantly enhanced removal efficiency compared to pristine biochar and maintained good stability under environmental conditions.

It is concluded that synergistic adsorption provides an effective strategy to transcend the intrinsic limitations of single-component biomass materials. By rationally integrating multiple mechanisms, such as physical adsorption, ion exchange, complexation, and redox reactions, high-performance adsorbents with improved capacity, selectivity, and environmental adaptability can be achieved. This approach offers a promising pathway for the design of next-generation biomass-derived materials for efficient heavy metal remediation.

## 5. Conclusions

In the pursuit of sustainable and eco-friendly solutions for mitigating the adverse effects of heavy metal contamination, this comprehensive review has investigated the remarkable potential of biomass-based materials as efficient adsorbents. Through an in-depth analysis of numerous studies and experimental findings, it is evident that plant-based, animal-based, and microbial-based biomass adsorption materials, with the advantages of wide availability, renewability, low cost, and good environmental compatibility, offer promising avenues for the removal of heavy metal ions from aqueous solutions. The exploration of influencing factors such as pH, temperature, and adsorbent dosage has provided a scientific basis for optimizing adsorption processes and improving treatment efficiency in practical engineering. In terms of adsorption mechanisms, the four core adsorption mechanisms (physical adsorption, chemical adsorption, ion exchange adsorption, and multi-pathway synergistic effects) employed by biomass adsorption materials have been elucidated, highlighting the significance of electrostatic interactions, van der Waals forces, hydrogen bonding, and ion exchange processes in heavy metal ion removal. Therefore, biomass-based adsorbents demonstrate irreplaceable advantages and broad application prospects in heavy metal ion pollution control. Future research should focus on developing efficient and low-cost modification techniques to enhance material adsorption capacity and selectivity, and further investigate adsorption mechanisms and synergistic interaction patterns under complex environmental conditions to improve theoretical frameworks. These efforts will promote the industrialization and sustainable development of biomass materials in heavy metal ion pollution control, providing globally competitive green technological solutions for ecological governance.

## Data Availability

Not applicable.
